# Exploiting Exchange-Correlation Functionals’ Performance for Structure and Property Prediction of the NaAlP_2_O_7_ Solid Electrolyte Material

**DOI:** 10.3390/ma19091673

**Published:** 2026-04-22

**Authors:** Mashaole Stuart Mamabolo, Donald Hlungwani, Kemeridge Tumelo Malatji, Phuti Esrom Ngoepe, Raesibe Sylvia Ledwaba

**Affiliations:** Materials Modelling Centre, University of Limpopo, Private Bag X 1106, Sovenga 0727, South Africa; 201310346@myturf.ul.ac.za (M.S.M.); donald.hlungwani@ul.ac.za (D.H.); kemeridge.malatji@ul.ac.za (K.T.M.)

**Keywords:** solid electrolytes, sodium ion batteries, stability, exchange-correlation functionals

## Abstract

First-principles calculations based on density functional theory (DFT) are a powerful tool in data-oriented materials research. The choice of approximation for the exchange-correlation functional is crucial, as it strongly affects the accuracy of DFT calculations. This study compares the performance capabilities of three approximations on the energetics, mechanical and electronic properties, and crystal structure of NaAlP_2_O_7_, which is an insulator with a wide band gap that suppresses its electronic conductivity. Two of these approximations are based on Perdew–Burke–Ernzerhof (PBE) generalized gradient approximation (GGA) and the other on the strongly constrained and appropriately normed (SCAN) meta-GGA. We explore these materials as a contribution to the development of new solid electrolytes (SEs) for sodium-ion batteries (NIBs), which have the potential to mitigate challenges related to lifecycle, safety, and low ionic conductivity. The performance of these batteries largely emanates from the extraordinary demand for high-performing energy storage technologies. This study revealed that PBEsol accurately predicted lattice parameters that closely aligned with experimental values. However, r2SCAN provided the most reliable predictions of the structural and electronic properties of the NaAlP_2_O_7_ solid electrolyte compared to PBE and PBEsol. Findings demonstrated that the material is structurally, mechanically, electronically, and thermodynamically stable, but exhibits vibrational instability, which may scatter ions and reduce ionic conductivity due to the presence of imaginary frequencies. Our results highlight the importance of selecting appropriate functionals for solid electrolyte DFT computations. The r2SCAN functional appears to be a promising choice for calculating NaAlP_2_O_7_ properties.

## 1. Introduction

The demand for new and advanced energy storage technologies has increased recently, driven by the rapid growth in the development of electronic devices, electric vehicles (EVs), and stationary power storage [1,2,3]. Sodium-ion batteries (NIBs), compared to lithium-ion batteries (LIBs), have attracted interest in the energy storage sector owing to their advantages, such as their low cost, safety, sodium abundance, and better electrochemical performance than LIBs [4,5,6,7]. However, NIBs face formidable challenges with low energy density [8], slow diffusion rate at the solid electrode during cycling, and large volume expansion compared with lithium-ion batteries [9], which play a role as setbacks that hinder the overall performance of NIBs. Currently, NIBs use liquid electrolytes, which benefit from high ionic conductivity (in the order of 10^−1^ S/m) [10]. However, these liquid electrolytes face challenges such as flammability due to thermal runaway [11], leakage due to the liquid boiling, leading to pressure buildup [12], a limited electrochemical stability window, and a short lifespan [13]. These issues raise safety concerns, particularly for large-scale applications, such as EVs and stationary power stations. Researchers have developed solid electrolytes (SEs) as promising alternatives to commercial liquid electrolytes to address these challenges in next-generation batteries [14].

Sodium superionic conductors (NASICONs) are promising solid electrolyte materials with a three-dimensional framework that allows the movement of ions through defects/vacancies within the crystal structure, leading to a higher ionic conductivity and exhibiting superior chemical and thermal stability properties [15,16]. The universally explored NASICON-type SE Na_3_Zr_2_Si_2_PO_12_ (NZSP) detailed by Goodenough et al. exhibits an excellent ionic conductivity of 6.7 × 10^−4^ S cm^−1^ at room temperature, which depends on the open three-dimensional channel for quick Na^+^ migration [17,18]. NZSP also demonstrates high chemical and thermal stability, which are essential for stable battery operation [14]. However, the assembled Na cells of NZSP demonstrate limited cycling stability (≤75% capacity retention after 100 cycles) [19]. The poor solid–solid contact and the insulating phases that form in the material lead to poor cyclic performance [20]. Additionally, the synthesis of NZSP is challenging because of the formation of second phases attributed to the volatilization of sodium and phosphorus, causing a change in composition during high-temperature processing. Extensive investigation has been dedicated to other NASICON-type Ses, such as Li_1.5_A_l0.5_Ge_1.5_(PO_4_)_3_, which exhibited a poor ionic conductivity of 5.4 × 10^−5^ S cm^−1^ at room temperature [21]. The NASICON-type SE material sodium aluminum pyrophosphate (NaAlP_2_O_7_) is a three-dimensional crystal framework structure, having the potential to exhibit high ionic conductivity due to its unique structural architecture which allows the transport of Na ions [22,23]. NaAlP_2_O_7_ material contains monovalent sodium, and due to its smaller ionic radii (in the range of 1.18–1.32 Å) than Li, it diffuses swiftly, resulting in high-rate capabilities. However, the NaAlP_2_O_7_ structure has few vacant sites for Na ions, which leads to moderate ionic conductivity. Therefore, incorporating NaAlP_2_O_7_ material with heterovalent dopants will create more vacant sites/defects, which will act as wider Na-ion pathways, leading to elevated ionic conductivity, which will improve the battery’s overall performance. The Al atoms are coordinated with six O atoms forming an AlO_6_ building block and the P atoms are coordinated by four O atoms forming the tetrahedral PO_4_. The structure contains a P_2_O group formed of two PO_4_’s which share corners to form a P-O-P bridge. The bond length and angle of this bridge play a key role in the material’s flexibility and vibrational properties. The Na atoms occupy the interstitial sites and are coordinated by eight O atoms to form a polyhedral group. The high number of Na ions and their irregular coordination are essential for its capabilities, as ion migration is one of the applications of SEs. The bond distance between the Na and O atoms and the energy barrier in this site are determined by the geometry of the Na-ion movement.

In this study, density functional theory (DFT) is employed to predict the different properties of SE materials. The exchange-correlation functionals, such as the Perdew–Burke–Ernzerhof (PBE) generalized gradient approximation (GGA) [24], the PBEsol (revised for solids) [25], and the r2SCAN meta-generalized gradient approximation (meta-GGA) functionals [26], have been employed to investigate the structural, electronic, mechanical, and thermodynamic properties of different SE materials due to their ability to predict accurate results. GGA functionals are renowned for striking a balance between computational efficiency and accuracy. However, GGAs often underestimate band gap energies for some materials, limiting their accuracy in predicting electronic properties [27]. The LDA functional underestimates the lattice parameters and bond length of the atoms in the crystal structure, while PBE overestimates them. Shang et al. reported that PBEsol predicted the structural properties of Li_2_CO_3_ in better agreement with experimental data than the PBE and LDA [28] functionals. Meta-GGAs, while more computationally expensive than GGAs, often offer greater accuracy by incorporating the kinetic energy density, leading to better predictions for solid and molecular properties [29,30]. Liu et al. demonstrated that the r2SCAN functional predicted the cell volumes, cohesive energy, and mechanical properties accurately compared to the LDA, PBEsol, and PBE functionals, while the PBEsol functional was superior in predicting the thermal expansion coefficients [31].

In this study, the capabilities of PBE, PBEsol, and r2SCAN to cater for accurate validation of NaAlP_2_O_7_ properties are investigated. This study employs DFT methods to calculate the mechanical and electronic properties of NaAlP_2_O_7_ materials due to their influence on the safety, performance, and lifespan of SEs. The exchange-correlation functional that offers the most accurate results will be used to unearth the electronic, mechanical, electrochemical, thermal, and vibrational properties of pyrophosphate-based NaAlP_2_O_7_ SEs in NIBs. These findings will lay a foundation for exploring the impact of various dopants on NaAlP_2_O_7_ SEs to mitigate the conductivity challenge while ensuring that the above-mentioned properties are not compromised, as they contribute to the overall performance of the battery.

## 2. Materials and Methods

In this section, the computational techniques employed to investigate the properties of NaAlP_2_O_7_ are explained in detail. All density functional theory (DFT) [32] calculations were performed using the Vienna Ab initio Simulation Package (VASP) [33].

### 2.1. Exchange Correlation Functionals

The exchange-correlation functional is an important approximation in DFT, which has a direct impact on the accuracy of the calculated properties. The currently proposed methods vary in terms of computational cost and accuracy. The local density approximation (LDA) is derived from the isotropic electronic density of a gaseous system [34]. The applicability of the LDA functional falls short in systems with varying charge densities [35]. The description of such systems is significantly improved with the introduction of the gradient of the local energy density. This is the generalized-gradient approximation (GGA) successfully parameterized by Perdew, Burke, and Ernzerhof (PBE). More accurate total energies and structural property predictions are expected with the inclusion of the charge density gradient [36]. This exchange-correlation functional yields relatively acceptable results from molecular systems with notable variation in electron densities to bulk materials with slowly changing electron densities. Nevertheless, it was shown that the performance of GGA-PBE can be improved for materials with densely populated atoms through the readjustment of the correlation term. The GGA functional revised for solids (GGA-PBEsol) can yield considerable improvements for equilibrium properties such as lattice parameters, mechanical, surface, phonon, and vibrational properties [37]. Despite the success of this modification of the GGA exchange functionals in a variety of material properties, for electronic properties, particularly for systems with strongly correlated electrons, sophisticated functionals are necessary. Perdew, Kurth, Zupan, and Blaha formulated a relatively robust functional by adding a kinetic energy density term to the local electron density and its gradient exchange-correlation functional called meta-GGA. This allows the incorporation of the density curvature to yield a more accurate exchange-correlation energy. The widely utilized meta-GGA exchange-correlation functional was parameterized by Tao, Perdew, Staroverov, and Scuseria (TPSS). The localization of d orbitals is notably achieved with meta-GGA functionals, particularly the strongly constrained and appropriately normed (SCAN) functional, which offers a more improved description of electronic and magnetic properties. However, a well-converged electronic density requires a high description of the reciprocal space, which is computationally demanding. The restored regularized SCAN (r2SCAN) functional was developed to achieve comparable accuracy at a relatively realistic computational cost [38,39,40].

### 2.2. Computational Details

The NaAlP_2_O_7_ structure used in this work is constructed from structural information (lattice parameters, space groups, etc.) obtained from experiments. All the calculations were performed with the Vienna ab initio simulation package (VASP) within the materials exploration and design analysis (MedeA) environment. Projector-augmented wave (PAW) [30] pseudopotentials were used for all exchange-correlation functionals to efficiently handle the core electrons. The damped molecular dynamics electronic minimization algorithm was able to find the ground-state electronic density of the NaAlP_2_O_7_ structure for all of the r2SCAN calculations. Moreover, a damping parameter of 0.4 fs was sufficient for achieving the ground-state electronic density. The electronic minimization was carried out by utilizing the Blocked-Davidson algorithm for the PBE and PBEsol calculations. The NaAlP_2_O_7_ structures were fully optimized using a plane wave basis set with a cut-off energy of 510.00 eV and *k-mesh* parameters of 4 × 4 × 3. The ground-state energy was reached when the change in total energy of the system was less than 10^−6^ eV. A denser sampling was chosen for density of states (DOS), band structures, and phonon dispersion calculations. For accurate calculation of these properties, the cut-off energy and k-points were set to 700 eV and 5 × 5 × 4, respectively. The NaAlP_2_O_7_ structure was fully optimized when the forces between the atoms were less than 1 × 10^−2^ eV/Å. The conjugate-gradient (CG) optimization scheme was utilized to determine atomic positions that minimize the total energy of the NaAlP_2_O_7_ system. A strain of 0.005 was applied on the NaAlP_2_O_7_ structure for the calculation of mechanical properties. The calculations utilized 168 and 36 bands for occupied and unoccupied orbitals, respectively. The phonon dispersion curve was predicted using the phonon code [41] as implemented in the MedeA [42] software.

## 3. Results and Discussions

### 3.1. Structural Analysis

#### 3.1.1. Structural Properties

**Figure 1:**
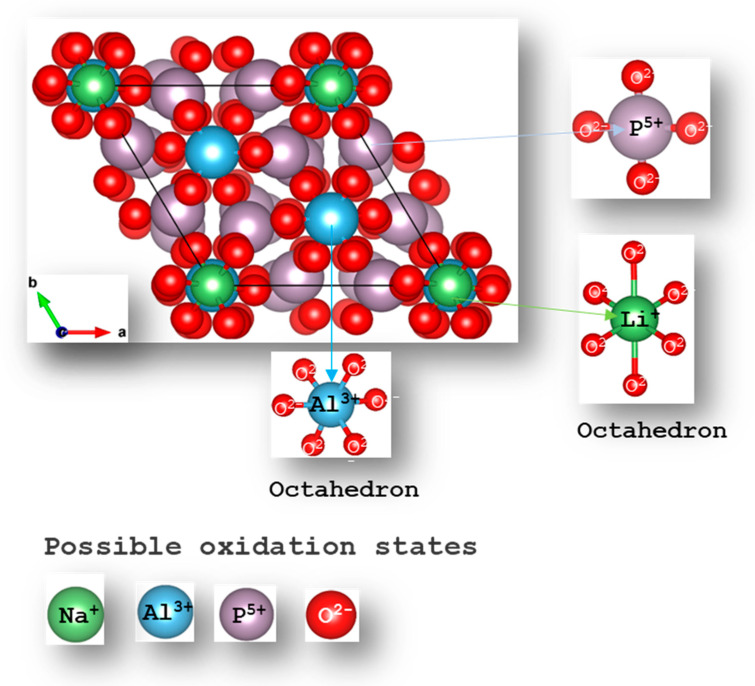
The crystal structure of a sodium aluminum pyrophosphate compound contains 44 atoms. The phosphate atoms form a tetrahedral coordination with the oxygen atoms; the aluminum and sodium ions form an octahedral coordination with oxygen.

The NaAlP_2_O_7_ crystal structure was optimized by allowing the cell volumes, shape, and atomic positions to relax using PBE, PBEsol, and r2SCAN exchange-correlation functionals. The lattice parameters and formation energies of the ground-state structure displayed in Figure 1 were obtained and examined. NaAlP_2_O_7_ is made up of 44 atoms and was found to crystallize in the monoclinic space group P_21_/c. The structure consists of corner-sharing P_2_O_7_ diphosphate groups and AlO_6_ octahedra, forming a three-dimensional framework composed of NaO_7_ polyhedra and AlO_6_ octahedra and P_2_O_7_ diphosphate units. Figure 1 illustrates the atomic arrangement of the NaAlP_2_O_7_ structure using the ball-and-stick and polyhedral views.

**Figure 2:**
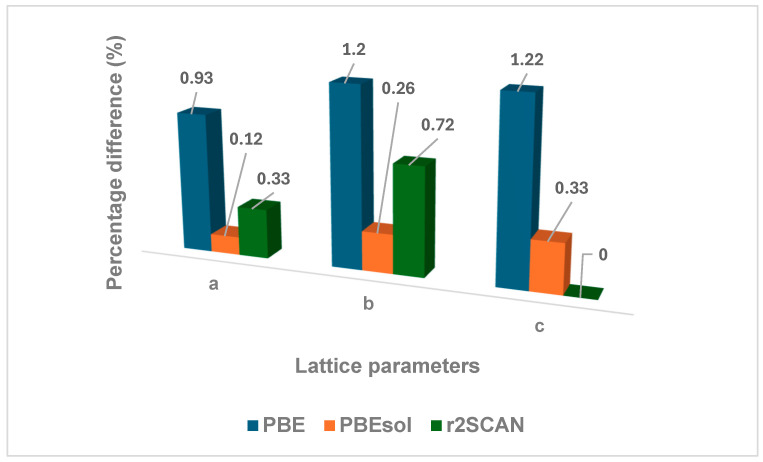
The percentage difference between the *a*, *b*, and *c* lattice parameters obtained in this work and the experimental values (Table 1) of the NaAlP_2_O_7_ structure.

The lattice parameters describe the smallest repeating units of a crystal structure, which is essential for understanding how materials behave under different conditions, such as variations in applied pressure, operating temperature, and stress, as well as for identifying the crystal structure [43]. Table 1 presents the lattice parameters, volumes, and densities of the NaAlP_2_O_7_ crystal structure calculated using PBE, PBEsol, and r2SCAN exchange-correlation functionals, compared with the experimental data reported by Taher et al. [44]. Figure 2 illustrates the percentage differences between the calculated lattice parameters of the NaAlP_2_O_7_ structure using the PBE, PBEsol, and r2SCAN exchange-correlation functionals compared to the experimental data. The lattice parameters calculated with the PBE functional show a large percentage difference, which illustrates that the PBE functional overestimates the lattice parameters of NaAlP_2_O_7_. This could lead to incorrect predictions of essential structure properties, such as the thermal expansion and the stability of various phases of the material under conditions such as differing pressure. The r2SCAN functional yielded percentage differences of 0.33% and 0.72% to experimental data for the a and b lattice parameters, respectively. The c parameter compared extremely well, with a percentage difference of 0% to experimental values. Notably, the PBEsol functional shows the best agreement with the experimental data, compared to the PBE and the r2SCAN functionals. Therefore, the PBEsol is the most suitable exchange-correlation functional for determining the lattice parameters of NaAlP_2_O_7_ solid electrolytes (SEs), which can contribute to reliable insights essential for developing sodium-ion batteries (NIBs) with enhanced performance.

**Figure 3:**
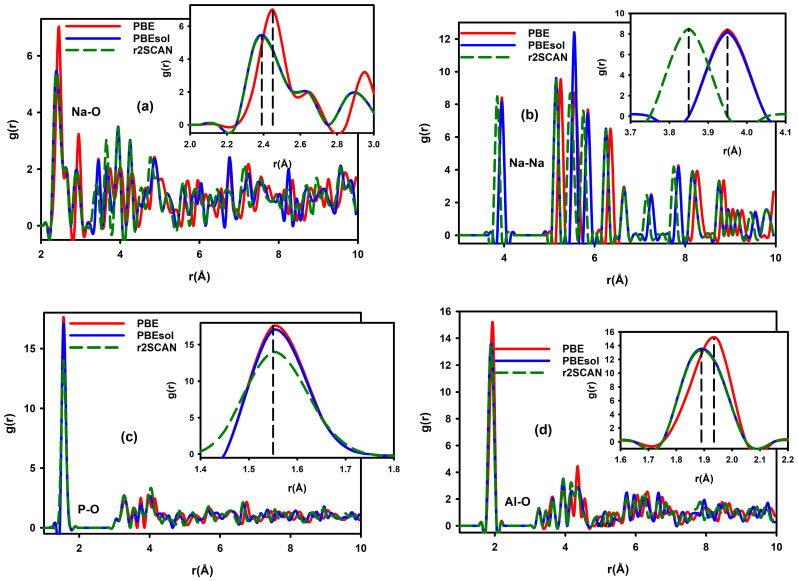
The bond lengths of (**a**) Na–O, (**b**) Na–Na (**c**) P–O, and (**d**) Al–O interactions in NaAlP_2_O_7_ are illustrated using radial distribution functions (RDFs) calculated from ground-state structures obtained with the PBE, PBEsol, and r2SCAN exchange-correlation functionals.

The RDF graphs in Figure 3a–d show the highest likelihood of finding an atom at a certain distance from a reference atom within a given separation range. The first peak in the RDF plot corresponds to the average distance to the nearest neighbouring atoms, which depicts the bonding distance of the interaction. The PBE first-nearest-neighbour RDF peak differs from the PBEsol and r2SCAN first-nearest-neighbour RDF peaks illustrated in Figure 3 for all interactions. This illustrates the differences in the magnitudes of the bonding distances between PBE, r2SCAN, and PBEsol for the NaAlP_2_O_7_ material. Table 2 shows the bonding distances obtained from the RDF plots of the NaAlP_2_O_7_ structure illustrated in Figure 3. The bond distance of the Na–Na interaction was found to be 3.962 Å, 3.912 Å, and 3.899 Å, obtained with the PBE, PBEsol, and r2SCAN exchange-correlation functionals, respectively. The bond length affects the shape and size of the channels through which ions migrate within the material. Narrow channels may slow down ion movement, resulting in low ionic conductivity. The Na–Na bonding distance represents the maximum migration path for Na^+^ conductivity in the material. As such, a shorter bond length reduces the energy barriers for ion migration, resulting in higher ionic conductivity. The PBE functional shows the highest bonding distance for all the interactions (Na–Na, Na–O, P–O, and Al–O). The bond lengths determined by the r2SCAN and PBEsol functionals are in good accord with a percentage difference of less than 1.3% for all the interactions (Na–Na, Na–O, P–O, and Al–O). Therefore, in line with the lattice parameters in Table 1, the PBEsol and r2SCAN functionals should yield bonding distances that are closer to experimental results.

**Figure 4:**
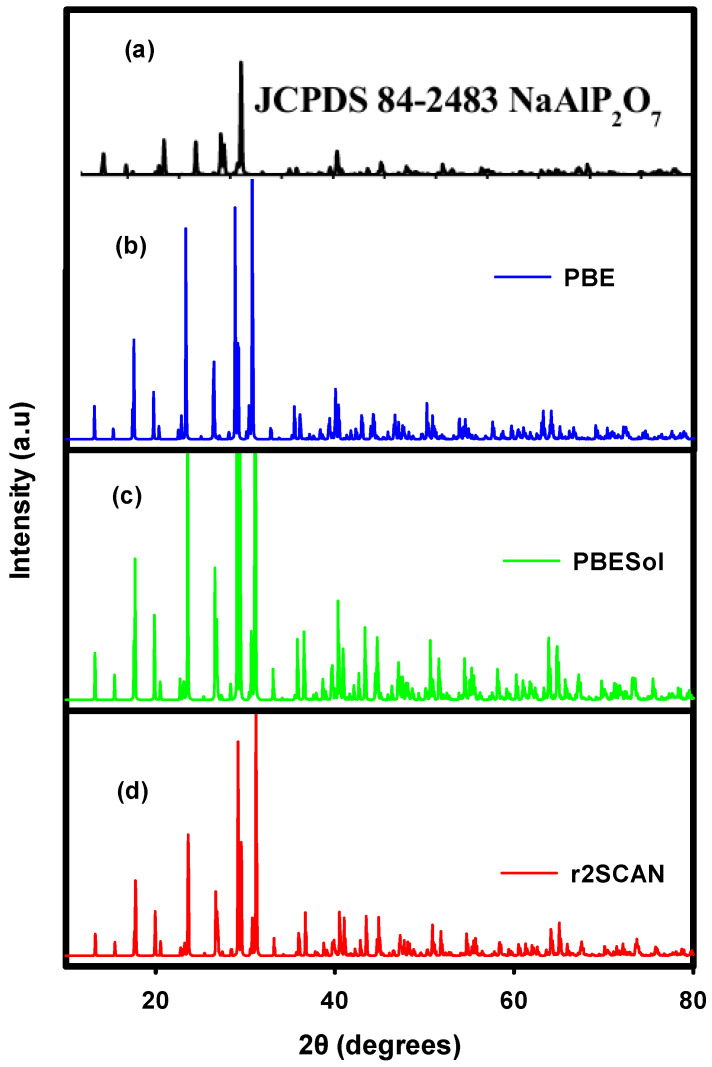
The XRD patterns of the NaAlP_2_O_7_ crystal structure: (**a**) the experimental structure [47] compared with the theoretical structure optimized using the (**b**) PBE, (**c**) PBEsol, and (**d**) r2SCAN exchange-correlation functionals.

The X-ray diffraction (XRD) patterns of NaAlP_2_O_7_ determined from its ground-state structures obtained with PBE, PBEsol, and r2SCAN exchange-correlation functionals, compared with that of experiments, are illustrated in Figure 4. Figure 4a compares well with the XRD patterns shown in Figure 4b–d, which are indexed to a monoclinic crystal structure with a space group of P2_1/c. The XRD patterns were calculated using a copper wavelength of 1.540 Å and indexed at various diffracting angles. The peaks diffracted along the 212 planes are observed at different diffraction angles for all of the structures, optimized with PBE (29.45°), PBEsol (29.37°), and r2SCAN (29.07°). This can be attributed to the different bond distances between the elements in these compounds obtained by different functionals. As such, the choice of the exchange-correlation functional greatly influences the quality and reliability of the results for this SE material. Additionally, the variations in the peak intensities demonstrated in Figure 4 further evidence the difference in the description of lattice properties with the three exchange-correlation functionals. The different peak intensities suggest a varying degree of crystallinity of the ground-state structures obtained with the three functionals. Furthermore, the calculated XRD patterns possess comparable width since they were generated from bulk structures of the same size, which is in line with the Debye–Scherrer equation [45,46]:

(1) $$D\;=\;\frac{K\lambda }{\beta cos(\theta )}\;$$

where D is the crystallite size, k=0.9 is a shape factor, λ is the X-ray wavelength, β is the full width half maxima (FWHM), and θ is the diffracting angle.

#### 3.1.2. Mechanical Properties

**Figure 5:**
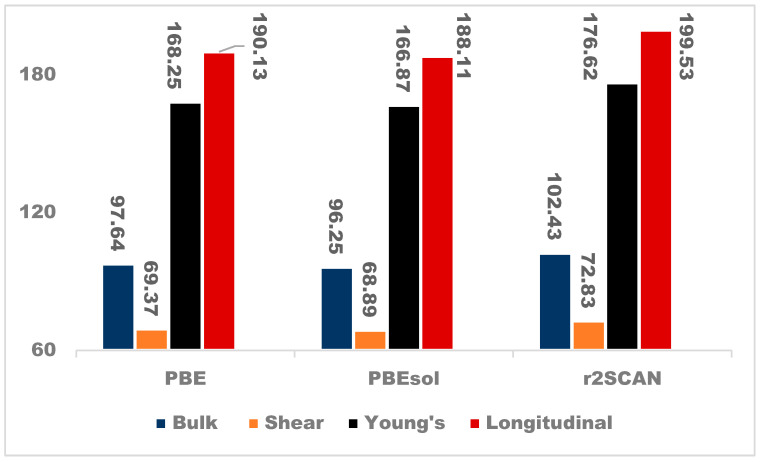
Elastic moduli (GPa) for NaAlP_2_O_7_ structures calculated using various functionals.

The investigation of the impact of the exchange-correlation functional on the mechanical properties of NaAlP_2_O_7_ was also evaluated, as illustrated in Table 3 and Figure 5. Table 3 depicts the elastic constants (C_ij_s) of NaAlP_2_O_7_ calculated with the PBE, PBEsol, and r2SCAN exchange-correlation functionals. There is currently no experimental data reported on the C_ij_s of the NaAlP_2_O_7_ material. The elastic constants were calculated under a strain of 0.005 applied isotopically to obtain a better mechanical response. For monoclinic crystals to be considered mechanically stable, they must satisfy the stability criteria defined by the Equations (A1)–(A7), illustrated in Appendix A.

Substitutions of the $C_{ij}s$ from Table 3 into Equations (A1)–(A7) satisfy all the stability criteria. Therefore, the three exchange-correlation functionals predict that the NaAlP_2_O_7_ material is mechanically stable. The mechanical stability is essential for maintaining structural integrity during charging and discharging of the secondary battery. The majority of the $C_{ij}$ constants are comparable, with slight differences. Particularly, the $C_{15}$, $C_{23}$, $C_{35}$, $C_{46}$, and $C_{66}$ constants of the three exchange-correlation functionals show notable differences. For example, the $C_{15}$ constant determined with the PBE, PBEsol, and r2SCAN functionals was found to be 4.52, 2.24, and −2.16, respectively. The PBEsol functional is designed to improve the accuracy of equilibrium properties, such as bulk moduli and lattice constants, by satisfying the second-order expansion of the gradient for local spin density and exchanged energy density.

The elastic modulus is also a very important mechanical property for assessing performance, and hence its proper description is of paramount importance. The elastic modulus describes the elastic deformation of a material under applied force [48], which plays a crucial role in the durability and performance of solid electrolyte (SE) batteries. Elastic properties are categorized into bulk modulus, shear modulus, Young’s modulus, and longitudinal modulus. The bulk modulus describes the uniform compression of a material in all directions when pressure is applied [49,50]. A material with a higher bulk modulus (100–150 GPa) indicates resistance to changes in volume.

The shear and bulk modulus are crucial in solid electrolyte batteries (SEBs) as they indicate a measure of a material’s resistance to cracking and deformation [51] under operating pressure (while maintaining constant volume), which affects the safety and lifecycle of the battery [50,52,53]. The bulk modulus of NaAlP_2_O_7_ was determined by PBE, PBEsol, and r2SCAN, illustrated in Figure 5, as 97.64 GPa, 96.25 GPa, and 102.43 GPa, respectively. The same trend is observed in the shear modulus, in which the PBESol has the lowest modulus (68.89 GPa) and the r2SCAN shows the highest modulus (72.83 GPa). The values of the Young’s and longitudinal moduli calculated with the PBE functional are between the values calculated with the PBESol and r2SCAN functionals. This trend is consistent for all of the elastic moduli. In general, oxide-containing solid electrolyte materials are brittle. Similarly, the Pugh’s ratio calculated with the PBE, PBESol, and r2SCAN functionals predicts that the NaAlP_2_O_7_ structure is brittle [54,55]. The values of the Pugh’s ratio determined with the three functionals are 1.41, 1.40, and 1.42 for the PBE, PBESol, and r2SCAN functionals, respectively. Their percentage difference is less than 2%, indicating that the three functionals yield comparable results for the elastic moduli. The elastic modulus is critical in studies of a material’s response to external stresses. A higher bulk modulus ensures structural integrity under applied loads, indicating greater resistance to volume changes under operating pressure [56]. Similarly, a higher shear modulus means the material resists bending and twisting, which can lead to fractures or cracks, and it can prevent the formation of Na dendrites because of the higher shear during battery operation. Batteries operate under varying temperatures and pressures, requiring rigid materials design and fabrication to avoid compression and stretching. The higher Young’s and longitudinal moduli obtained with r2SCAN, as displayed in Figure 5, reveal that the NaAlP_2_O_7_ solid electrolyte (SE) material is stiff and resists deformation under applied pressure [57]. However, factors such as the crystal’s structure and the temperature, pressure [58], defects, and chemical composition can affect the elastic properties of SE materials. A higher bulk modulus is one of the criteria used to determine whether the material is brittle [56].

#### 3.1.3. Electronic Properties

**Figure 6:**
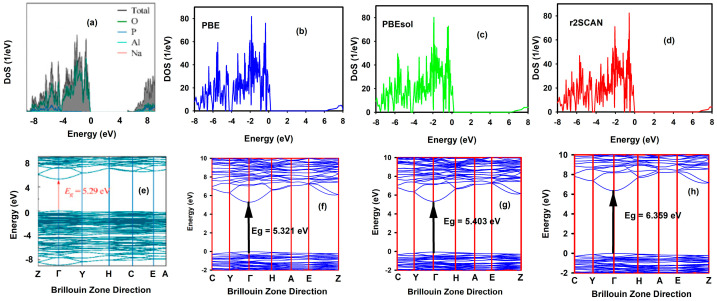
The total density of states (TDOS) of NaAlP_2_O_7_: (**a**) experimental [60] compared with calculations using the (**b**) PBE, (**c**) r2SCAN, and (**d**) PBEsol exchange-correlation functionals and the band structure of NaAlP_2_O_7_: (**e**) experimental findings [44] versus that of the (**f**) PBE, (**g**) PBEsol, and (**h**) r2SCAN exchange-correlation functionals.

The electronic properties of a material describe the behaviour and interactions of electrons within the material, which influence ionic conductivity, electronic conductivity, safety, and the electrochemical stability window. These properties play a pivotal role in determining how materials respond to electric fields, heat, and light. A wider band gap is desirable in solid electrolyte (SE) batteries, as it can improve their stability and safety by suppressing electronic conductivities and reducing the likelihood of unwanted chemical reactions and short circuits [59]. Figure 6 shows the total density of states (TDOS) of the NaAlP_2_O_7_ crystal structure calculated using the PBE (band gap of 5.321 eV), PBEsol (5.403 eV), and r2SCAN (band gap of 6.359 eV) exchange-correlation functionals implemented in VASP. The obtained band gap energies from all three functionals indicate insulating behaviour. The TDOS calculated by r2SCAN exhibits a wider band gap, meaning that electrons in the valence band (VB) require more energy to transition to the conduction band (CB). The PBE functional shows the smallest energy gap, with a significant energy difference of 1.038 eV compared to that of the r2SCAN functional. Therefore, the choice of functional is very sensitive to the electronic properties of materials such as NaAlP_2_O_7_.

Additionally, the electronic property analysis of NaAlP_2_O_7_ was also evaluated with band structure diagrams, illustrated in Figure 6e–h, which describe the behaviour of electrons occupying different energy levels within a material. It gives a pictorial description of the valence band, the highest occupied energy level at zero temperature, and the conduction band, the unoccupied energy level above the valence band at absolute zero. The band gap energy of 5.321 eV for NaAlP_2_O_7_, calculated using the PBE functional, agrees with the band gap energy of 5.29 eV reported by Zhang et al. [60]. Zhang et al. also employed the PBE functional in their DFT prediction of the band gap of NaAlP_2_O_7_, which aligns with our predictions. The PBESol functional demonstrates a band gap of 5.403 eV for NaAlP_2_O_7_, which is 0.08 eV higher than the band gap computed with the PBE functional. Hence, the PBE and PBESol functionals yield similar predictions of the electronic properties of NaAlP_2_O_7_. In comparison with the PBE and PBESol functionals, the r2SCAN functional overestimates band gap energies, which is a consequence of its enhanced treatment of electron interactions. The smaller band gaps obtained with the PBE and PBEsol functionals can be attributed to the inherent poor description of localized electrons by the two functionals. The r2SCAN meta-GGA functional includes crucial information about the orbitals in the materials through the kinetic energy density, which provides a better description of the localized electronic environments in the material than the PBE and PBEsol functionals. As such, the most accurate description of electronic interaction is expected from the r2SCAN functional rather than the PBE and PBESol functionals. However, the band gap determined by the r2SCAN functional can be overestimated in comparison with the true band of the material, in line with the observations of Kothakonda et al. It was reported that r2SCAN overestimated the band gap energy of the material, which was found to be higher than the experimental value [29]. This overestimation can be traced to inaccuracies in how r2SCAN handles an electron’s interaction with its charge within the material. These errors cause an overestimation of the energy levels associated with the valence and conduction bands, leading to an increased estimated band gap for NaAlP_2_O_7_.

#### 3.1.4. Thermodynamic Properties

For a material to be considered thermodynamically stable, it should exhibit negative formation energy. The formation energies of the NaAlP_2_O_7_ structure were calculated using the PBE, PBEsol, and r2SCAN exchange-correlation functionals using the formula in Equation (2). The energy of formation of NaAlP_2_O_7_ was found to be −2641.60 kJ/mol, −2747.15 kJ/mol, and −3003.22 kJ/mol with the PBE, PBEsol, and r2SCAN exchange-correlation functionals, respectively. All the functionals suggest that NaAlP_2_O_7_ is thermodynamically stable, evidenced by the negative energy of formation. This indicates that the NaAlP_2_O_7_ structure has a strong tendency to avoid decomposition and phase transition, making it a stable material. The higher energy of formation values calculated with the PBE functional can be linked to the overestimation of the lattice parameters, which leads to weaker atomic bonds and consequently to slightly higher energies. The PBEsol functional systematically yields high energy of formation values due to its capabilities of properly describing properties such as lattice parameters and bulk moduli. The r2SCAN functional produced the most thermodynamically stable structure with the lowest energy of formation. The r2SCAN functional is expected to yield an energy of formation that is closer to the experimental values due to its efficiency in characterizing local environments such as the double bond in O_2_ molecules. This is poorly defined in the PBE and PBEsol functionals, resulting in a large difference in the energy of formation calculation, in line with Equation (2). Therefore, the r2SCAN functional can be the most effective approach in predicting the ground-state interaction energy, quantified by formation energy, with relatively low computational demands.

(2) $$E_{F}\left({NaAlP}_{2}O_{7}\right)=E_{T}\left({NaAlP}_{2}O_{7}\right)-\left(E_{ref}\left(Na\right)+E_{ref}\left(Al\right)+2E_{ref}\left(P\right)+\left(\frac{7}{2}\right)E_{ref}\left(O_{7}\right)\right),$$

where $E_{F}\left({NaAlP}_{2}O_{7}\right)$ is the energy of formation for the compound [61], $E_{T}\left({NaAlP}_{2}O_{7}\right)$ is the total energy of the compound, and $E_{ref}$ is the reference energy of each constituent of NaAlP_2_O_7_.

**Figure 7:**
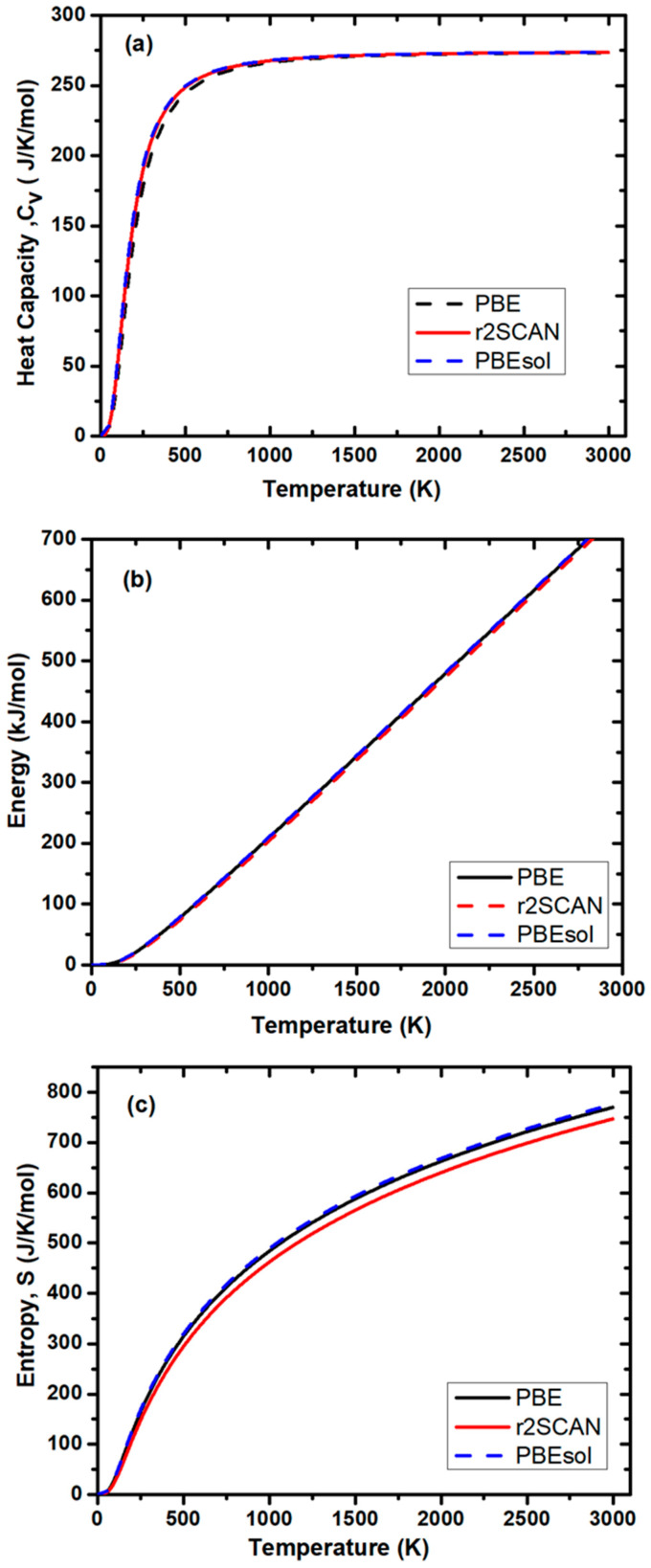
(**a**) Vibrational heat capacity at constant volume, (**b**) internal energy, and (**c**) entropy as a function of temperature for NaAlP_2_O_7_ structures.

An increase in temperature significantly influences ion diffusion and mobility within solid electrolytes (SEs), as vibrating ions gain more kinetic energy, leading to higher battery capacity. Elevated temperatures can also accelerate electrochemical reactions at the electrode–electrolyte interface, improving reaction kinetics and enabling higher current densities and capacity. However, at very high temperatures, capacity may decrease due to increased side reactions, such as electrode corrosion and electrolyte decomposition, which can degrade battery performance. At low temperatures (near absolute zero), the heat capacity of NaAlP_2_O_7_ increases slowly because only low-energy vibrational modes of the atoms are excited. As the temperature rises, more vibrational modes become accessible, resulting in a gradual increase in heat capacity. This low-rate increase in heat capacity is attributed to the growing number of excited vibrational modes with temperature. At elevated temperatures, the heat capacity approaches a constant value, as shown in Figure 7a. Table 4 shows the heat capacity values at room temperature and 333 K, indicating that the battery can operate at low temperatures. This value corresponds to the energy required to excite all vibrational degrees of freedom of the atom in the material. The three functionals demonstrate a similar trend of heat capacity, with a slight difference noted between 250 and 750 K. The PBE functional yields results that deviate slightly from the PBEsol and r2SCAN functionals. This is attributed to the overinflated lattice parameters resulting from the softening of the low-frequency acoustic modes. Since soft modes are easily affected by temperature, this is more visible at lower temperatures (250–500 K). The PBEsol and r2SCAN functionals show good correlation to the heat capacities since they both yield acceptable lattice parameters. Lattice parameters possess significant influence on low frequency modes, which dominate heat capacities.

The internal energy of the system, calculated with three functionals, PBE, PBEsol, and r2SCAN, increases linearly with temperature, as shown in Figure 7b. This trend can be attributed to the increase in vibrational energy of the atoms in the NaAlP_2_O_7_ structure. As the temperature rises, the kinetic energy of the atoms increases due to their dynamic vibrations. At higher temperatures, the atoms in NaAlP_2_O_7_ gain sufficient energy to move freely within the material, leading to higher ionic conductivity. Among the functionals, PBEsol predicts higher energy, followed by r2SCAN and then PBE. The parallel lines in the figure indicate that the energy difference between the functionals remains consistent across the temperature range. However, the PBE and PBEsol functionals demonstrate a subtle increase in the internal energy of the system in comparison with the r2SCAN functional. In PBE, the softer phonon is generally inaccurate, which leads to inaccurate zero-point energies. This is expected to improve with the PBEsol and r2SCAN functionals. The r2SCAN functional generally yields accurate phonons due to the inclusion of the kinetic term in its description.

Entropy in a system measures the level of disorder or randomness among atoms within a material, reflecting their arrangement. Higher entropy promotes the formation of more ionic pathways for ionic migration, as increased vacant sites and defects improve ionic conductivity by allowing atoms to move more easily. This makes entropy a key factor in designing batteries for various applications, such as electric vehicles (EVs) and electronic devices. Figure 7c shows the relationship between entropy and temperature predicted with three different exchange-correlation functionals, PBE, PBEsol, and r2SCAN. At higher temperatures, thermal energy helps ions overcome energy barriers, enabling them to move more freely within the electrolyte. This increased mobility raises entropy, indicating a greater level of disorder. However, very high temperatures can generate heat during battery operation, potentially reducing the battery’s lifespan and raising safety concerns. The insert in Figure 7c demonstrates how low temperatures affect the entropy of SEs. At temperatures between 0 and 30 K, entropy is much lower, but it sharply increases at 20 K, showing that ions gain enough energy to migrate within the NaAlP_2_O_7_ SE material. The PBE and PBEsol functionals predicted comparable entropy with increasing temperature, which varies from those predicted with the r2SCAN functional. This can be linked to the lack of the dispersion in PBE and PBEsol compared to the r2SCAN functional, which captures the intermediate range of the dispersion.

**Figure 8:**
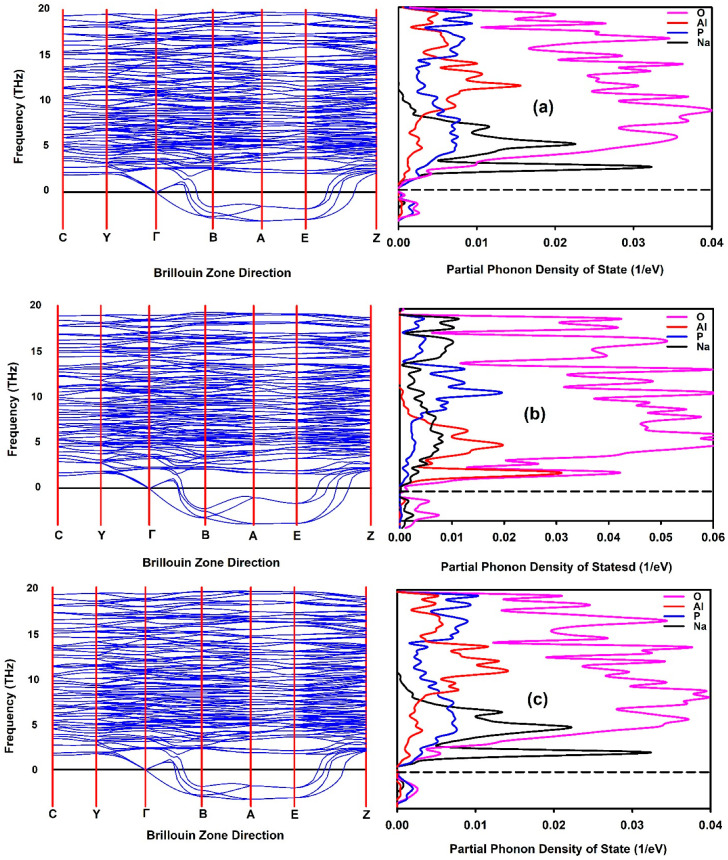
Phonon dispersion curves and partial phonon density of states of O (pink), Al (red), P (blue), and Na (black) for NaAlP_2_O_7_ calculated using (**a**) PBE, (**b**) PBEsol, and (**c**) r2SCAN exchange-correlation functionals.

The quantum mechanical concept of “phonons” describes a specific type of vibrational motion in which lattice oscillations occur uniformly at the same frequency. Phonon dispersions in SEs are crucial for understanding material behaviour, including ionic transport, which influences ionic conductivity, thermal properties, and material design approaches. Phonons are categorized into two types: optical and acoustic modes. Optical modes, characterized by positive frequencies, are indicative of vibrational stability, while acoustic modes, with negative frequencies, indicate vibrational instability. Figure 8 shows the phonon dispersion curves of the NaAlP_2_O_7_ structure, calculated using the PBE, PBEsol, and r2SCAN exchange-correlation functionals implemented in VASP. Soft modes are observed in all phonon dispersion curves, indicating structural instability [62] in the NaAlP_2_O_7_ structure calculated with these functionals. As such, phonon calculations in NaAlP_2_O_7_ are not particularly affected by the choice of the functionals, between PBE, PBEsol, and r2SCAN. The instability in the NaAlP_2_O_7_ structure is attributed to the atomic vibrations of the Na, Al, P, and O atoms in the A, B, and E directions. The O and P atoms exhibit higher vibrational velocities in the A and E directions, as shown in the partial phonon density of states (DOS). Additionally, the O and P atoms are located closer to the Fermi level compared to the Na and Al atoms. Based on the obtained phonon dispersion curves, it is not possible to determine which functional is the most suitable for evaluating the vibrational stability of the NaAlP_2_O_7_ structure.

## 4. Conclusions

This study successfully investigated the properties of NaAlP_2_O_7_ using different density functional theory exchange-correlation functionals, such as PBE, PBEsol, and r2SCAN. The PBEsol functional was found to yield lattice parameters that are in close agreement with experimental data. Consequently, the PBEsol functional can also be a viable option for calculating mechanical properties within acceptable computational demands. The NaAlP_2_O_7_ structure was found to be mechanically stable, satisfying all stability criteria for a monoclinic crystal. Wider band gap energies were observed, showing the insulating behaviour of NaAlP_2_O_7_. The PBE and PBEsol functionals were also found to underestimate the band gap of the NaAlP_2_O_7_ solid electrolyte material. As a result, the r2SCAN functional could be a better approach for electronic structure analysis at a considerable computational cost. The three functionals demonstrate that the NaAlP_2_O_7_ structure is thermodynamically stable, which is essential for the lifecycle of sodium-ion batteries. However, the r2SCAN functional also predicted a more negative energy of formation than the PBE and PBEsol functionals. As such, the calculation of formation energies of the NaAlP_2_O_7_ solid material benefits significantly from the inclusion of the kinetic energy density in the r2SCAN-meta-GGA functional. The choice of the exchange-correlation functional was also found to be very important in the calculation of the internal energy and entropy of the NaAlP_2_O_7_ solid electrolyte material. The r2SCAN was found to be a suitable option for calculation of these properties due to its proper description of soft modes and inclusion of intermediate-range dispersion. The three functionals (PBE, PBEsol, and r2SCAN) predict that the NaAlP_2_O_7_ structure is vibrationally unstable, demonstrated by the negative frequencies in the phonon dispersion curves, likely due to atomic oscillation speeds. As such, structural modification through doping can be sought for improving its vibrational stability. Overall, the r2SCAN exchange-correlation functional demonstrated superior accuracy in predicting most of NaAlP_2_O_7_’s properties, making it a reliable choice for modelling solid electrolyte materials. However, the PBEsol functional can also play a pivotal role in accelerating analysis of NaAlP_2_O_7_’s equilibrium properties, especially in situations where a bigger simulation structure is required. These findings provide valuable insights into developing NIBs with solid electrolytes, enhancing battery safety and performance across numerous applications.

## Figures and Tables

**Table 1:** The lattice parameters (a, b, c), angles (*α*, *β*), volume, and density of the NaAlP_2_O_7_ structure calculated using the PBE, PBEsol, and r2SCAN exchange-correlation functionals, compared with experimental results.

PBE	PBEsol	R2SCAN	Experimental
*a* = 7.264 Å	*a* = 7.206 Å	*a* = 7.173 Å	*a* = 7.197 Å
*b* = 7.800 Å	*b* = 7.687 Å	*b* = 7.652 Å	*b* = 7.707 Å [44]
*c* = 9.409 Å	*c* = 9.326 Å	*c* = 9.295 Å	*c* = 9.295 Å
β = 111.630°	β = 111.652°	β = 111.603°	β = 111.701°
α = 90.000	α = 90.000	α = 90.000	-
V = 495.294 Å^3^	V = 480.077 Å^3^	V = 474.359 Å^3^	-
ρ = 3.003 Mg/m^3^	ρ = 3.098 Mg/m^3^	ρ = 3.135 Mg/m^3^	-

**Table 2:** Bond lengths of the structural building blocks in NaAlP_2_O_7_ calculated using the PBE, PBEsol, and r2SCAN exchange-correlation functionals.

Interaction	PBE	PBEsol	r2SCAN
Na–Na	3.962	3.912	3.899
Na–O	2.452	2.386	2.416
P–O	1.509	1.514	1.508
Al–O	1.867	1.850	1.861

**Table 3:** The elastic constants of the NaAlP_2_O_7_ structure were calculated using different exchange-correlation functionals.

C_ij_	PBE (GPa)	PBEsol (GPa)	r2SCAN (GPa)
C_11_	226.04	214.15	221.11
C_12_	49.37	51.03	53.08
C_13_	54.42	54.12	54.88
C_15_	4.52	2.24	−2.16
C_22_	146.39	124.81	137.61
C_23_	47.35	58.53	63.69
C_25_	−0.76	−7.50	−9.79
C_33_	204.02	199.91	219.85
C_35_	4.92	3.44	4.15
C_44_	80.05	82.83	88.54
C_46_	5.78	−1.14	−1.54
C_55_	63.61	64.36	63.16
C_66_	61.40	72.21	76.79

**Table 4:** The heat capacity and temperature values calculated using the PBE, PBEsol, and r2SCAN exchange-correlation functionals.

Functionals	Temperature (K)	Heat Capacity (J/K/mol)
PBE	298 333	200 211
PBEsol	298 333	211 222
r2SCAN	298 333	208 219

## Data Availability

The original contributions presented in this study are included in the article. Further inquiries can be directed to the corresponding authors.

## Abbreviations

The following abbreviations are used in this manuscript:

DFT	Density Functional Theory
GGA-PBE	Generalize Gradient Approximation Perdew–Burke–Ernzerhof
GGA-PBESOL	Generalize Gradient Approximation Perdew–Burke–Ernzerhof Revised for Solids
Meta-GGA r2SCAN	Generalize Gradient Approximation Range-separated Hybrid functional
LDA	Local Density Approximation
VASP	Vienna Ab initio Simulation Package
PAW	Projector-Augmented Wave
LIB	Lithium-Ion Batter
NIB	Sodium-Ion Battery
SE	Solid Electrolyte
SEB	Solid Electrolyte Battery
EV	Electric Vehicle
Na	Sodium
Al	Aluminum
P	Phosphorus
O	Oxygen
NZSP	Sodium Zirconium Silicon Phosphate
NASICON	Sodium Superionic Conductor
DOS	Density Of States
TDOS	Total Density of States
PDOS	Phonon Density of States
D	Crystallite Size
XRD	X-ray Diffraction
FWHM	Full Width Half Maxima
RDF	Radial Distribution Functional
VB	Valence Band
CB	Conduction Band
C_ij_	Elastic Constant
GPa	Gigapascal
eV	Electron Volts

## Appendix A

The mechanical stability conditions for a monoclinic system, as outlined by the Born criteria, are expressed as [54,63]:

(A1) $$(C_{44}C_{66}-C_{46}^{2})>0$$

(A2) $$(C_{22}+C_{33}-2C_{23})>0$$

(A3) $$(C_{35}C_{55}-C_{35}^{2})>0$$

(A4) $$[C_{11}+C_{12}+C_{33}+2(C_{12}+C_{13}+C_{23})]>0$$

(A5) $$\left[C_{22}\left(C_{33}C_{55}-C_{35}^{2}\right)+2C_{23}C_{25}C_{32}-C_{23}^{2}C_{55}-C_{25}^{2}C_{33}\right]>0$$

(A6) $$C_{22}+C_{33}-2C_{23}>0$$

(A7) $$C_{11}>0,\;C_{22}>0,\;C_{33}>0,\;C_{44}>0,\;C_{55}>0,\;and\;C_{66}>0$$
